# Research highlights for issue 4: Predicting the evolutionary response of populations to climate change

**DOI:** 10.1111/eva.12158

**Published:** 2014-04-17

**Authors:** Britt Koskella

Given the increasingly unpredictable weather patterns associated with global climate change, a key aim of current research is to predict whether and how populations will be able to respond. Major advances are being made through a combination of natural, long-term studies and experimental approaches, for example by experimentally manipulating the environment and measuring phenotypic and/or genetic/genomic change. The first issue of *Evolutionary Applications* this year (Volume 7, Issue 1), under the lead of guest editors Andrew Hendry and Juha Merilä, brought together perspectives from a number of researchers on the leading edge of climate change research. This included multiple synthetic reviews discussing the challenges of teasing apart evolutionary change from more ‘plastic’ responses to environmental perturbation, as well as reviews of the current empirical evidence for and against key theoretical predictions. Overall, the volume highlights that, although data on the evolutionary potential of populations in response to climate change are accumulating, we are still far from where we hope to be in terms of predictive ability, especially given the complexity of most environments (Merilä and Hendry 2014).

Such biological and environmental complexity can be disentangled through the use of experimentally manipulated microcosms, and these studies provide a powerful tool for differentiating correlation from causation and investigating interactive, synergistic effects among multiple factors. Researchers in this field are moving beyond tests of increasing temperature to examine more complex factors, including environmental fluctuations and multiple abiotic or biotic stressors (Jeffs and Leather 2014; Vasseur et al. 2014). Recent work by Jennifer Lau and collaborators manipulated both the atmospheric CO_2_ concentration and the competitive environment of experimental *Arabidopsis thaliana* populations. They found that, while populations showed little direct response to increased CO_2_, there were strong indirect effects of the abiotic environment on the population level response to competitor-mediated selection (Lau et al. 2014). This type of indirect effect of climate change on populations would be overlooked in studies focused solely on climate-specific adaptations. Similarly, the effect of climate change at the community level can often be more striking than effects on a single population or species (Bailey et al. 2014). Sarah Evans and colleagues examined adaptation of soil bacterial communities after a decade of drying and rewetting stress and found that community composition was significantly altered relative to plots experiencing normal precipitation, especially in favor of those taxa exhibiting increased stress tolerance (Evans and Wallenstein 2014). The outcome of adaptation to changing environmental conditions can also differ in very meaningful ways among populations or genotypes. Romain Gallet and coauthors recently evolved replicate populations of *Escherichia coli* under extreme shifts in pH over 2,000 generations and found adaptation to the new pH in all replicates but evidence for niche width expansion in only two of the four replicates. This latter finding emphasizes the importance of incorporating the evolution of plasticity into predictions regarding climate-mediated adaptation and also highlights the very different evolutionary trajectories that populations may take in response to the same selection pressure (Gallet et al. 2014).

Although these experimental approaches offer important insight to the processes underlying climate-mediated evolutionary change, an understanding of whether a population *can* respond does not clearly translate into an understanding of whether it *will* respond. As such, there is still great need for long-term studies from natural populations. Katie Becklin and colleagues have examined changes in leaf physiology across seven conifer species by comparing samples preserved in middens (debris piles) from five time points since the last glacial maximum. Their data suggest physiological adaptations of leaves to climate change, such as decreased stomatal conductance, were influenced by evolutionary history but were not the primary determinant of shifts in community composition (Becklin et al. 2014). Finally, combining both natural and experimental datasets can offer a unique perspective on our ability to predict the effects of climate change. Sean Menke and collaborators have compared the results of long-term experimental data with those from natural populations to demonstrate that, although ant community composition has shifted along an elevational gradient over time in the natural populations, there has been relatively little change over the course of experimental warming (Menke et al. 2014). This work highlights the potential limitations of microcosm studies in isolation, given the biological complexity of populations and communities over space and time. However, these limitations can be overcome in part with biologically meaningful replication of experimental plots. This is nicely demonstrated by a recent study of wetland seed banks from multiple latitudes across two continents in which species diversity of communities from southern latitudes were found to be less affected by experimental warming than those from more northern latitudes (Baldwin et al. 2014).

Together, these studies demonstrate the need to incorporate both biotic and abiotic complexity into models and empirical studies in order to fully understand the resilience of populations and communities in the face of climate change. The impressive range of recent approaches, from experimental manipulation of multiple factors, to seminatural studies of communities spanning a wide range of abiotic conditions, to tests of species-level adaptations from preserved specimens across millions of years, has allowed great strides in our understanding of both if and how populations might respond to the increasingly unpredictable environment.
